# Spontaneous coronary artery dissection as a threshold disorder of coronary wall integrity

**DOI:** 10.3389/fcvm.2026.1887304

**Published:** 2026-07-06

**Authors:** Zhongwei Liu, Jun Wang

**Affiliations:** 1 Department of Cardiology, Shaanxi Provincial People’s Hospital, Xi’an, China; 2 Department of Anesthesiology, Shaanxi Provincial Cancer Hospital, Xi’an, China

**Keywords:** coronary wall integrity, extracellular matrix, fibromuscular dysplasia, intramural hematoma, precision cardiovascular medicine, pregnancy-associated myocardial infarction, spontaneous coronary artery dissection, vascular smooth muscle cell

## Abstract

Spontaneous coronary artery dissection (SCAD) is an increasingly recognized cause of non-atherosclerotic acute myocardial infarction, predominantly affecting young and middle-aged women and patients during pregnancy or the postpartum period. Although major progress has been made in clinical recognition, angiographic diagnosis, conservative management, and extracoronary vascular screening, the molecular basis of SCAD remains incompletely understood. Here, we develop a provisional, evidence-integrating framework that conceptualizes SCAD as a threshold disorder of coronary wall integrity rather than as a variant of plaque rupture or a purely intimal tear-driven event. The available evidence indicates that the final common pathological event is intramural hematoma formation and true-lumen compression, whereas the threshold for this event is shaped by inherited susceptibility, extracellular matrix architecture, vascular smooth muscle cell and fibroblast regulation, vascular tone, local hemostatic containment, systemic arteriopathy, and reproductive or stress-related hemodynamic load. We critically evaluate inside-out and outside-in models of SCAD initiation, common and rare genetic architecture, the ambiguous role of fibromuscular dysplasia, pregnancy-associated SCAD, antithrombotic controversies, and emerging strategies for mechanism-informed risk stratification. We also define what is established, what remains controversial, and what should not yet be translated into routine practice. Finally, we propose a research roadmap centered on multiomic prospective cohorts, coronary wall models, high-resolution imaging phenotypes, reproductive vascular biology, and pragmatic clinical trials. The resulting taxonomy is intended to organize current evidence, identify priorities for mechanistic validation, and potentially support precision diagnosis, individualized antithrombotic decisions, reproductive counseling, genetic testing, and prevention of recurrence.

## 1 Introduction: reframing SCAD as a threshold disorder of coronary wall integrity

Spontaneous coronary artery dissection (SCAD) occupies a paradoxical position in contemporary cardiovascular medicine. It is now widely recognized as an important cause of acute coronary syndrome and myocardial infarction in young and middle-aged women, in individuals with few conventional atherosclerotic risk factors, and in patients presenting during pregnancy or the postpartum period. The American Heart Association scientific statement established SCAD as a distinct clinical entity requiring diagnostic and management principles that differ from plaque rupture myocardial infarction (1). The European position paper further emphasized that SCAD is not simply an angiographic curiosity but a disease category with specific diagnostic pitfalls, acute management dilemmas, and long-term follow-up needs (2).

Despite this clinical maturation, SCAD remains mechanistically immature compared with atherosclerotic coronary artery disease. The JACC state-of-the-art review captured the rapid expansion of clinical knowledge while also highlighting persistent gaps in recurrence prediction, pregnancy counseling, genetic evaluation, and post-SCAD symptom burden (3). Earlier single-center cohort work had already shown that SCAD patients were predominantly women, often younger than patients with atherosclerotic MI, and faced recurrent events over long-term follow-up (4). Population-based and registry studies then demonstrated that the apparent rarity of SCAD partly reflected under-recognition, incomplete angiographic classification, and failure to consider non-atherosclerotic mechanisms in women with ACS (5).

This Review develops a provisional threshold framework in which SCAD is best understood as a disorder of coronary wall integrity. The final common pathway is obstruction of the true lumen by blood within the arterial wall, but the threshold for this event is set by interacting determinants: inherited variation affecting extracellular matrix and vascular wall cell regulation; local mechanical stress, arterial curvature, and vascular tone; tissue-mediated hemostatic responses; reproductive and hormonal-hemodynamic states; and systemic arteriopathies such as fibromuscular dysplasia (FMD). Earlier clinical reviews helped consolidate the definition, angiographic recognition, and management vocabulary of SCAD (6). The next step is to integrate those clinical observations with genetic, pathological, and translational evidence into a biological framework that can be tested in future mechanistic studies.

The threshold model avoids three oversimplifications. First, SCAD is not a plaque rupture variant, because the culprit lesion is not atherothrombosis over a disrupted lipid-rich plaque. Second, SCAD is not usually a Mendelian connective tissue disorder, because clearly pathogenic rare variants explain only a minority of cases. Third, SCAD is not synonymous with coronary FMD, because extracoronary FMD is common but incomplete, and available coronary histology does not establish FMD as a universal proximate lesion. The value of a threshold model is that it preserves heterogeneity at initiation while explaining convergence at the level of intramural hematoma and true-lumen compression. Throughout this Review, the model is used as an organizing construct for evidence synthesis and hypothesis development rather than as a settled causal paradigm.

## 2 Clinical and angiographic foundations: defining the wall-based lesion

SCAD is defined by spontaneous separation of the coronary arterial wall in the absence of atherosclerotic plaque rupture, trauma, or iatrogenic manipulation. The clinical consequence is myocardial ischemia caused by compression of the true lumen by a false lumen or intramural hematoma. Angiographically, SCAD may appear as multiple radiolucent lumens and contrast staining, long diffuse smooth narrowing, abrupt caliber change, or complete occlusion. The Saw angiographic classification was pivotal because it made the field aware that the most frequent phenotype may be diffuse narrowing rather than a classic double lumen (7).

Diagnostic difficulty remains central to SCAD biology because angiography is a luminogram, whereas SCAD is fundamentally a wall disease. Ambiguous lesions may be misclassified as vasospasm, atherosclerotic stenosis, embolism, or microvascular disease unless operators actively consider the possibility of SCAD. A contemporary diagnostic framework therefore requires wall-aware interpretation, selective use of OCT or intravascular ultrasound, and careful avoidance of procedures that might propagate a fragile wall lesion (8). Practical diagnostic algorithms have helped clarify when SCAD should be suspected and when intracoronary imaging can be justified in uncertain cases (9).

Several clinical facts are now established. SCAD predominantly affects women, often without conventional cardiovascular risk factors. It may occur in association with emotional stress, intense physical exertion, pregnancy or the postpartum period, migraine, FMD, systemic arteriopathy, and rarely monogenic connective tissue disease. Stable patients are usually managed conservatively because the dissected vessel frequently heals and because PCI can be technically challenging. Revascularization can be necessary in left main involvement, ongoing ischemia, hemodynamic compromise, or refractory ventricular arrhythmia, but PCI in SCAD carries a distinct risk of hematoma propagation, false-lumen wiring, side-branch compromise, and long stent requirements (10).

The implication is that SCAD should be conceptualized anatomically from the wall outward rather than from the lumen inward. A stenotic lumen is the clinical manifestation, but the pathological compartment is the media-adventitia structural unit. This distinction is not semantic. It shapes how clinicians interpret diffuse narrowing, why conservative management often succeeds, why repeat imaging may show healing, and why standard plaque-rupture ACS logic can be misleading.

## 3 Clinical modifiers and outcomes: why the threshold is crossed in selected patients

SCAD requires both vulnerability and context. A vulnerable coronary wall may remain clinically silent until mechanical, hormonal, autonomic, or hemodynamic demand exceeds its reserve. In a large Vancouver cohort, Saw and colleagues showed that SCAD is frequently associated with predisposing arteriopathies and precipitating stressors, including emotional and physical triggers (11). This observation should not be interpreted as a claim that stress alone causes SCAD. Rather, it supports a threshold concept in which acute exposures act on an already susceptible wall substrate.

Prospective registries have refined this picture. The Canadian SCAD cohort documented early clinical outcomes, angiographic characteristics, and associated arteriopathies in a large adjudicated population (12). Longer follow-up from the same cohort showed low mortality but meaningful recurrent MI, recurrent SCAD, persistent chest pain, and associations between adverse outcomes and factors such as FMD or genetic disorders (13). These findings support a disease model in which the acute obstruction may heal but the patient-level vascular vulnerability can persist.

The Australian-New Zealand SCAD cohort added contemporary data on predictors of major adverse cardiovascular events and recurrence (14). Importantly, this study linked outcomes with factors that have mechanistic relevance, including FMD, stroke history, oral anticoagulation at discharge, and aspirin plus ticagrelor use. These observations are not proof of causality, but they intensify the need to distinguish plaque-rupture ACS from a wall-bleeding syndrome.

Pregnancy-associated SCAD is an especially informative human stress-test of coronary wall reserve. The iSCAD Registry report identified clinical and reproductive variables associated with pregnancy-associated SCAD and confirmed that this subgroup can present with more severe ACS phenotypes (15). Counseling after SCAD must therefore be individualized rather than categorical, integrating residual left ventricular function, vascular imaging, genetic suspicion, blood pressure control, and patient values (16). Studies of survivors and nonsurvivors of pregnancy-associated SCAD have further emphasized that peripartum physiology can produce severe proximal, multivessel, or left-main presentations in a minority of patients (17).

Pregnancy-associated SCAD should not be reduced to a simple estrogen or progesterone story. Pregnancy and the postpartum period involve major shifts in plasma volume, cardiac output, vascular compliance, matrix turnover, coagulation, sympathetic tone, and hypertensive disorders of pregnancy. The threshold model accommodates these interacting forces without prematurely assigning a single endocrine mechanism. It also implies that future registries should collect detailed reproductive variables, assisted reproductive technology exposure, preeclampsia history, lactation status, blood pressure trajectories, and postpartum timing.

## 4 Intramural hematoma as the final common pathological event

The most useful way to organize SCAD mechanisms is to separate upstream determinants from the proximal pathological event. Proximally, SCAD is a disease of blood accumulating within the coronary arterial wall, compressing the true lumen and producing ischemia. Upstream, the route by which this accumulation begins may differ. The classic inside-out model proposes that an intimal tear permits luminal blood under arterial pressure to enter the media. The outside-in model proposes that bleeding begins within the arterial wall, potentially from mural microdisruption or small intramural vessels, producing an expanding hematoma that may or may not subsequently communicate with the lumen.

Histopathology provides the strongest constraint on overly simple theories. Systematic examination of coronary tissue and connective tissue ultrastructure in SCAD did not support increased vasa vasorum density, local inflammation, or coronary FMD as universal direct causes (18). This finding does not exclude these processes in individual cases, but it argues against presenting any one of them as the default mechanism. The common denominator remains intramural hematoma and true-lumen compression.

A vascular mechanics perspective helps reconcile inside-out and outside-in models. The coronary artery is exposed to cyclical bending, torsion, shear stress, and pulsatile circumferential strain. Segments with tortuosity, hinge points, or branch-related curvature may experience focal amplification of wall stress. Coronary tortuosity has been reported as highly prevalent in SCAD and may mark a mechanical environment in which modest material weakness becomes clinically relevant (19). Tortuosity is unlikely to be sufficient by itself, but it may interact with impaired matrix support, altered vascular tone, or local hemostatic failure.

This concept also explains why SCAD can heal. Unlike atherosclerotic plaque rupture, which leaves a diseased plaque substrate and a thrombogenic disrupted surface, SCAD often resolves as the hematoma resorbs and the wall remodels. Conservative treatment is therefore logical when coronary flow is preserved and ischemia is not ongoing. The success of conservative therapy does not mean that the initiating biology is benign; it means the acute obstruction is often dynamic and reversible if the hematoma does not propagate.

**Figure 1:**
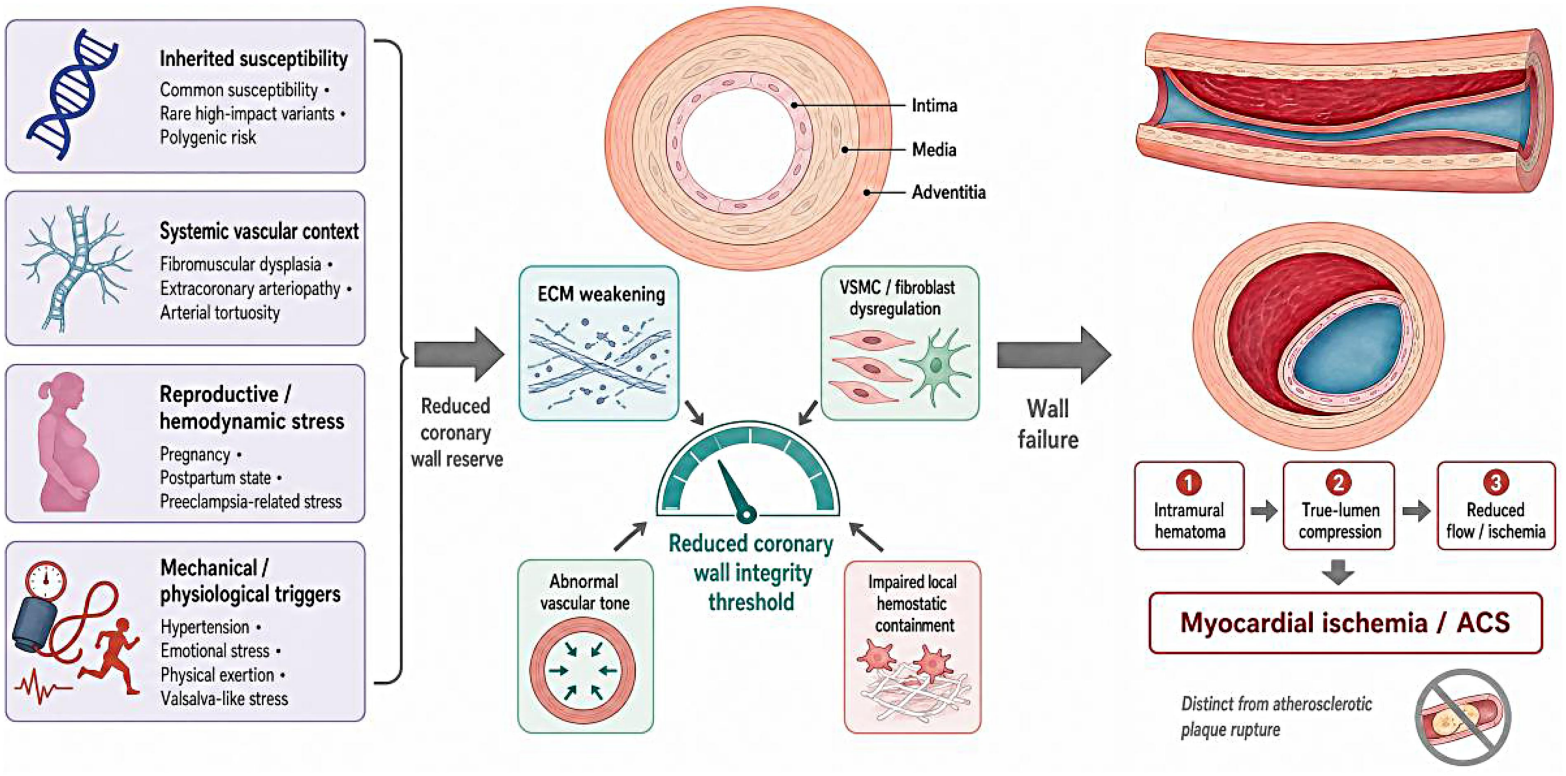
Spontaneous coronary artery dissection as a threshold disorder of coronary arterial wall integrity. The schematic summarizes the central mechanistic framework of this Review. Multiple predisposing contexts and triggers—including inherited susceptibility, systemic vascular arteriopathy, reproductive or hemodynamic stress, and acute mechanical or physiological triggers—converge to reduce coronary wall reserve. Within the vulnerable coronary arterial wall, extracellular matrix weakening, vascular smooth muscle cell and fibroblast dysregulation, abnormal vascular tone, and impaired local hemostatic containment collectively lower the threshold for arterial wall failure. Once this threshold is crossed, blood accumulates within the arterial wall as an intramural hematoma, leading to false-lumen expansion, true-lumen compression, reduced coronary blood flow, and myocardial ischemia or acute coronary syndrome. This model emphasizes SCAD as a non-atherosclerotic coronary wall failure syndrome that is mechanistically distinct from atherosclerotic plaque rupture. ACS, acute coronary syndrome; ECM, extracellular matrix; SCAD, spontaneous coronary artery dissection; VSMC, vascular smooth muscle cell. The framework is presented as an evidence-integrating model to guide mechanistic validation rather than as proof of a fixed causal sequence.

Taken together, the pathological and mechanical observations support a working threshold model in which patient-level susceptibility and acute stressors converge on a vulnerable arterial wall. As illustrated in Figure 1, predisposing context and triggers reduce coronary wall reserve, while matrix, vascular-wall-cell, tone, and local hemostatic modules lower the wall-integrity threshold and culminate in intramural hematoma, true-lumen compression, and ischemia. The figure presents these relationships as an integrative summary of current evidence rather than as a definitive causal sequence.

## 5 Genetic architecture: from monogenic mimics to polygenic wall vulnerability

Genetic research has transformed SCAD from a clinically defined arteriopathy into a disease with measurable, layered inherited susceptibility. The first major insight was the association of the PHACTR1/EDN1 locus with SCAD, connecting the disease to vascular tone, endothelial signaling, and pleiotropic arterial biology (20). This locus is particularly informative because it has divergent associations across vascular diseases, including SCAD, FMD, migraine, and atherosclerotic CAD. Such pleiotropy suggests that genomic context can influence different arterial phenotypes through distinct cell types, tissues, and mechanical environments.

Subsequent GWAS expanded the field beyond a single locus. Turley and colleagues identified susceptibility loci for SCAD and implicated arterial tissue expression and vascular biology (21). A separate genetic study identified chromosome 1q21.2 and additional loci influencing risk of both SCAD and myocardial infarction, reinforcing the idea that SCAD-related MI is genetically distinct from atherosclerotic MI (22). The strongest common-variant evidence now comes from the large genome-wide association meta-analysis by Adlam and colleagues, which identified multiple SCAD risk loci and implicated arterial integrity, extracellular matrix biology, vascular tone, and tissue-mediated coagulation (23).

The biological interpretation of GWAS results is crucial. GWAS loci do not identify deterministic causal genes in individual patients. They identify genomic regions that alter population susceptibility, usually through regulatory effects. The SCAD loci are enriched in arterial tissues and regulatory elements relevant to VSMCs and fibroblasts. Candidate loci near ECM1, ADAMTSL4, COL4A1/COL4A2, and F3 support the wall-integrity model more strongly than any single-pathway model. The field should therefore resist the temptation to describe SCAD as an endothelin disease, a collagen disease, or a coagulation disease. The genetic data point to convergence, not singularity.

Rare-variant studies add a second layer. Genome sequencing has shown that only a minority of SCAD patients carry clearly pathogenic or likely pathogenic variants in genes associated with connective tissue or vascular disorders (24). Exome and biobank analyses have also reported enrichment of disruptive variants in fibrillar collagen genes, with complementary experimental evidence supporting a relationship between collagen disruption and arterial dissection susceptibility (25). High-risk SCAD patients may carry an increased burden of rare variants in vascular connective tissue disorder genes, especially when presentation includes young age, recurrent disease, systemic arteriopathy, family history, or syndromic features (26).

Familial SCAD further illustrates the complexity of inheritance. Family-based sequencing can identify rare variants in selected pedigrees, but family clustering is not always explained by a single high-effect mutation (27). Polygenic risk analysis suggests that inherited susceptibility can be distributed across many loci and can contribute to both familial and apparently sporadic SCAD (28). Recent attention to COL3A1 variants underscores the importance of recognizing vascular Ehlers-Danlos-related phenotypes while avoiding the misconception that most SCAD is a classical collagen disorder (29). Additional cohort studies, including those from non-North American populations, are beginning to broaden the genetic architecture beyond the first wave of discovery studies (30).

The distinction between rare high-effect variants and common polygenic susceptibility should shape clinical practice. Universal broad genetic testing of every patient with typical sporadic SCAD is unlikely to be efficient and may generate variants of uncertain significance. Conversely, genetics referral is reasonable for younger patients, recurrent SCAD, multivessel dissections, extracoronary aneurysm or dissection, family history, syndromic physical features, severe pregnancy-associated SCAD, or unusual vascular findings.

**Figure 2:**
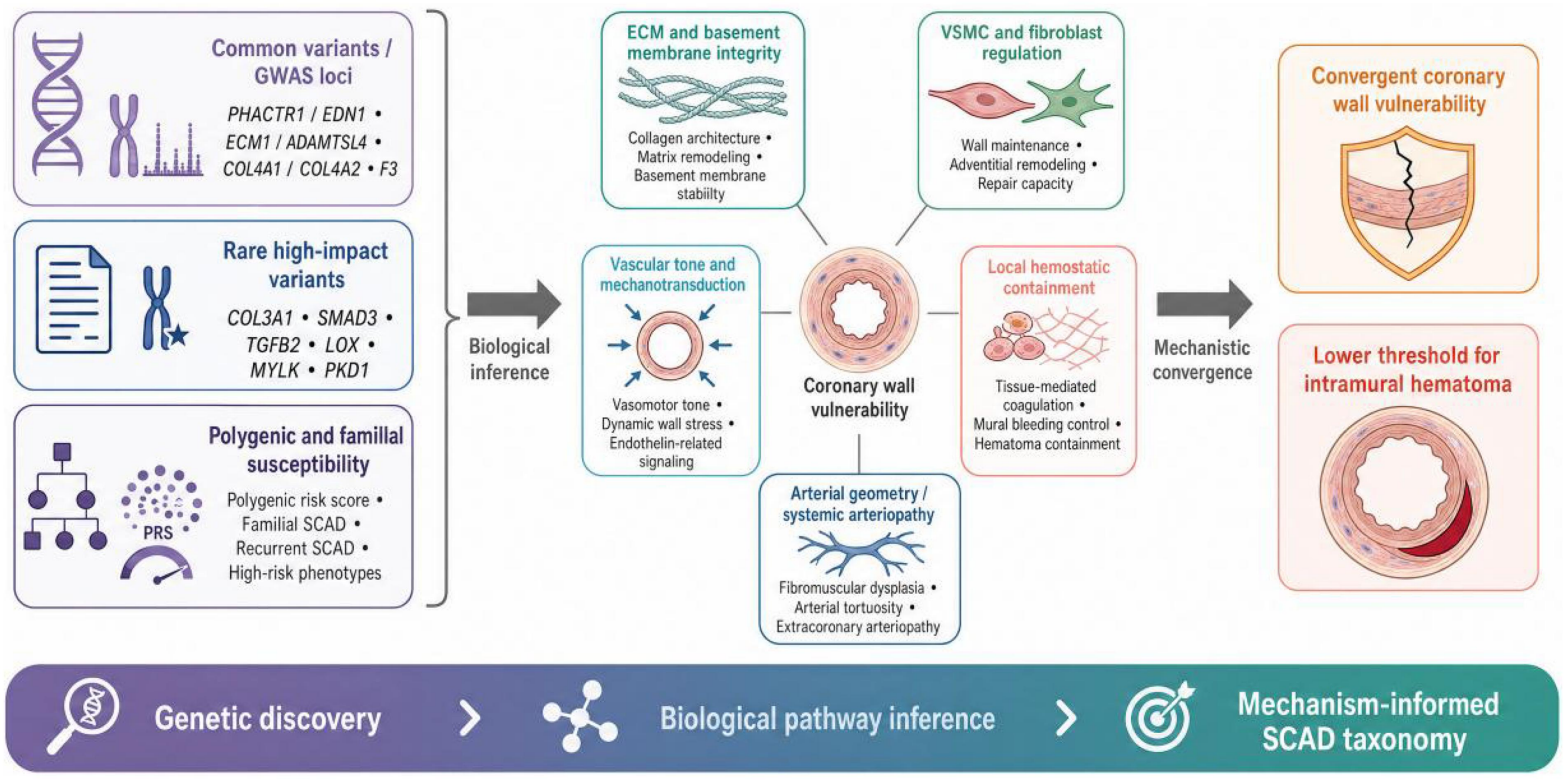
Genetic evidence converges on biological pathways that define coronary wall vulnerability in SCAD. The schematic illustrates how distinct layers of inherited susceptibility contribute to the mechanistic understanding of spontaneous coronary artery dissection (SCAD). Common variant and genome-wide association study signals, rare high-impact variants, and polygenic or familial susceptibility are shown as complementary evidence layers rather than deterministic causes. These genetic data converge on biological modules related to extracellular matrix and basement membrane integrity, vascular smooth muscle cell and fibroblast regulation, vascular tone and mechanotransduction, local hemostatic containment, and arterial geometry or systemic arteriopathy. Together, these pathways support the concept that SCAD arises from convergent coronary wall vulnerability and a reduced threshold for intramural hematoma formation. The lower panel summarizes the proposed interpretive progression from genetic discovery to biological pathway inference and, ultimately, to a mechanism-informed SCAD taxonomy. ECM, extracellular matrix; GWAS, genome-wide association study; PRS, polygenic risk score; SCAD, spontaneous coronary artery dissection; VSMC, vascular smooth muscle cell. Table 1 organizes representative genetic evidence by biological module and clinical interpretation, while preserving the distinction between population-level susceptibility signals and actionable high-risk variants. Candidate loci and genes are used to illustrate pathway-level interpretation and should not be read as validated patient-level causal assignments.

These genetic layers are therefore best interpreted as convergent biological evidence rather than as isolated gene lists or one-gene-one-disease explanations. As illustrated in Figure 2, common susceptibility loci, rare high-impact variants, and polygenic or familial risk map onto shared modules of extracellular matrix and basement membrane integrity, vascular wall cell regulation, tone and mechanotransduction, local hemostatic containment, and systemic arteriopathy. The arrows indicate biological inference and pathway convergence, not direct causation from any single locus.

**Table 1:** Genetic architecture of SCAD organized by biological mechanism.

Genetic layer	Representative examples	Biological module	Clinical interpretation	Caution
Common risk loci	PHACTR1/EDN1; ECM1/ADAMTSL4; COL4A1/COL4A2; F3	Vascular tone, matrix integrity, basement membrane biology, local hemostasis	Common variants support a distributed susceptibility architecture.	Do not treat common loci as deterministic patient-level diagnoses.
Rare high-impact variants	COL3A1; SMAD3; TGFB2; LOX; MYLK; PKD1	Connective tissue and vascular wall integrity	Useful for selected high-risk phenotypes and family counseling.	Most typical sporadic SCAD does not have a monogenic explanation.
Fibrillar collagen burden	Collagen gene-disrupting variants	ECM tensile strength	Supports matrix weakness as a plausible route to dissection susceptibility.	Rare-variant enrichment does not imply universal collagen disease.
Familial aggregation	Affected pedigrees, recurrent disease, high-risk vascular phenotypes	Shared inherited wall vulnerability	Supports family history assessment and selected genetics referral.	Familial disease may still be polygenic or multifactorial.
Polygenic susceptibility	SCAD polygenic risk scores	Distributed regulation of arterial wall biology	May eventually contribute to risk stratification.	Clinical thresholds are not yet validated.
Emerging regulatory candidates	Small-RNA processing and post-transcriptional regulators	Vascular gene regulation and biomarker biology	May connect inherited susceptibility with circulating molecular signatures.	Replication and functional validation are required.

## 6 Convergent biological mechanisms: matrix, wall cells, tone, hemostasis, and systemic arteriopathy

The strongest mechanistic interpretation of current evidence is not that SCAD is caused by a single pathway, but that multiple upstream processes reduce coronary wall reserve. Four local modules deserve emphasis: extracellular matrix and basement membrane organization; vascular wall cell phenotype; vascular tone and mechanotransduction; and local hemostatic containment. These modules are interdependent. VSMCs and fibroblasts synthesize and remodel matrix; matrix regulates cell phenotype and mechanosensing; vascular tone changes wall stress; and local coagulation determines whether mural bleeding remains microscopic or becomes a compressive hematoma.

Extracellular matrix biology is the most intuitive module. The arterial media and adventitia depend on collagen, elastin, proteoglycans, basement membrane components, and matrix-associated proteins to maintain tensile strength and elasticity. Rare collagen variants provide direct support in selected patients, whereas common-variant signals suggest that modest perturbations in matrix composition or remodeling may reduce the safety margin of the coronary wall under transient stress. This mechanism should be framed as reduced material reserve rather than as universal connective tissue disease.

VSMCs and fibroblasts form the cellular interface between inherited susceptibility and wall mechanics. VSMCs are responsible for contractile tone, matrix synthesis, response to injury, and structural maintenance of the media. Adventitial fibroblasts regulate matrix remodeling, mechanical coupling, and tissue repair. Enrichment of SCAD risk loci in arterial regulatory elements suggests that inherited susceptibility may act through altered gene expression in these cell types rather than through coding disruption alone. This is a major reason why future SCAD biology should include single-cell and spatial approaches in human arterial tissue when available, and mechanostimulated vascular wall models when tissue is not available.

Vascular tone bridges molecular biology and triggering physiology. Endothelin-related biology, adrenergic surges, vasoconstriction, and blood pressure elevation can increase circumferential stress and amplify focal loading at tortuous or branch-related segments. This rationale supports hemodynamic modulation as a plausible preventive strategy. It does not prove that vasospasm or hypertension alone causes SCAD, and it should not lead to a simplistic vasospasm model. A more precise formulation is that abnormal vascular tone and mechanical responsiveness can lower the threshold at which a vulnerable wall fails.

Local hemostatic containment is an emerging and underdeveloped module. Genetic signals implicating tissue-mediated coagulation suggest that the ability of the wall to contain microbleeding may be relevant. This does not mean that SCAD is a systemic clotting disorder. The relevant question is whether the coronary wall can rapidly seal microscopic mural injury. A wall that cannot contain a small bleed may convert a subclinical event into an expanding intramural hematoma. This distinction is central for therapeutic inference: systemic anticoagulation may be harmful in selected conservatively managed patients not because coagulation is irrelevant, but because local mural hemostasis may be protective once bleeding has begun.

Systemic arteriopathy is the fifth module because SCAD commonly coexists with abnormalities outside the coronary bed. Extracoronary vascular imaging has shown that FMD, aneurysm, dissection, and arterial tortuosity can be present in SCAD survivors, although severe multivessel disease is not universal (31). FMD consensus documents provide a framework for diagnosis and management of extracoronary disease (32). Earlier state-of-the-science work in FMD also emphasized that FMD itself is heterogeneous across vascular territories and cannot be reduced to a single radiographic label (33). The appropriate conclusion is that systemic arteriopathy may mark shared wall biology, shared geometry, or shared developmental susceptibility, but should not be treated as synonymous with SCAD.

## 7 Evidence hierarchy: what is established, what is plausible, and what remains fragile

A high-level mechanistic Review must grade evidence rather than summarize all findings at equal weight. The strongest evidence currently comes from large replicated genetic studies, prospective clinical registries with adjudicated lesions, and systematic pathology. These data establish that SCAD is genetically distinct from atherosclerotic CAD, that arterial wall integrity pathways are implicated, that intramural hematoma is the central pathological substrate, and that clinical outcomes differ from plaque rupture ACS.

Moderate evidence supports several modifiers. FMD and extracoronary arteriopathy are clearly associated with SCAD, but the causal direction and direct coronary-wall mechanism remain unresolved. Pregnancy and postpartum status are strongly associated with severe SCAD presentations, but the exact molecular mechanisms are inferred rather than directly demonstrated. Blood pressure and vascular tone are compelling because they have genetic and physiological support, but randomized preventive evidence remains limited. Coronary tortuosity is credible as a marker of mechanical vulnerability, but whether it is causal, compensatory, or epiphenomenal remains uncertain.

Fragile evidence includes several attractive but incompletely verified hypotheses: primary vasa vasorum rupture as the universal cause, local inflammation as the dominant driver, universal connective tissue ultrastructural abnormality, and direct extrapolation from systemic dissection syndromes to typical sporadic SCAD. These mechanisms may operate in subsets, but current evidence does not justify presenting them as general explanations. This caution is important because statements about recurrence, pregnancy, exercise, and medication affect patient quality of life.

Experimental evidence remains the major bottleneck. Unlike atherosclerosis, SCAD lacks widely accepted animal models, lesion-prone *in vitro* systems, or routinely available diseased human coronary tissue. Rare-variant mouse models support the plausibility of matrix weakness, but murine arterial biology and coronary mechanics differ from human SCAD. The field therefore needs models that reproduce not only arterial weakening but also intramural hematoma formation under pulsatile pressure, bending stress, and local hemostatic challenge.

Because the available literature spans consensus documents, angiographic studies, pathology, genetics, registries, biomarkers, and trial designs, the evidence should not be treated as uniform. As summarized in Table 2, the current evidence base is strongest for clinical definition, replicated genetic association, registry-based outcomes, and systematic pathology, whereas experimental modeling and some mechanistic modifiers remain less mature. Accordingly, the threshold framework functions best as a working model for organizing evidence and prioritizing validation studies.

**Table 2:** Evidence hierarchy linking SCAD mechanisms to study domains.

Evidence domain	Core contribution	Mechanistic inference	Evidence strength	Main limitation
Consensus and state-of-the-art reviews	Define SCAD as a distinct non-atherosclerotic ACS entity.	Clinical boundaries justify a disease model distinct from plaque rupture.	High for clinical consensus	Mechanistic causality is inferred rather than directly tested.
Angiography and intravascular imaging	Clarify diffuse narrowing, double lumen, intimal flap, and intramural hematoma phenotypes.	SCAD should be interpreted as a wall-based lesion, not only a luminal stenosis.	Moderate to high	Selective imaging and procedural risk limit uniform assessment.
Histopathology	Shows intramural hematoma and constrains universal theories of inflammation, FMD, or vasa vasorum rupture.	Supports heterogeneity at initiation and convergence at wall hematoma.	High for available tissue	Fatal and surgical specimens may overrepresent severe phenotypes.
Common-variant genetics	Identifies loci related to arterial integrity, matrix biology, vascular tone, and tissue hemostasis.	Supports distributed wall vulnerability rather than a single disease gene.	High for association	Causal genes and effector cell types require functional validation.
Rare-variant genetics	Finds high-impact variants in selected patients, especially connective tissue and vascular genes.	Defines clinically important high-risk subgroups.	Moderate	Low yield in unselected sporadic SCAD and frequent variants of uncertain significance.
Prospective clinical cohorts	Quantify early outcomes, recurrence, persistent symptoms, and associated arteriopathies.	Link wall vulnerability with long-term patient-level risk.	Moderate to high	Observational design leaves residual confounding.
Pregnancy-specific cohorts	Identify reproductive variables and severe phenotypes in pregnancy-associated SCAD.	Pregnancy can act as a physiological stress-test of coronary wall reserve.	Moderate	Mechanistic biospecimens and longitudinal vascular measures are limited.
Biomarker studies	Suggest molecular signatures distinguishing SCAD from atherosclerotic AMI.	Potential path to acute diagnosis and phenotyping.	Early	Requires large real-world validation.

## 8 Therapeutic and translational implications: from ACS extrapolation to mechanism-informed care

The threshold model has immediate translational consequences. If SCAD is treated as plaque rupture ACS, the default response is antithrombotic intensification and revascularization. If SCAD is treated as coronary wall failure with intramural hematoma, the default response becomes stabilization of the wall environment, avoidance of iatrogenic propagation, individualized antithrombotic decisions, blood pressure and heart-rate control, and long-term risk stratification. This does not mean that antithrombotics are never appropriate. Many SCAD patients present as ACS, some undergo PCI, and luminal thrombus can coexist. It means that the mechanism of ischemia is not necessarily thrombosis over a ruptured plaque.

Antiplatelet therapy is the most visible clinical controversy generated by the intramural hematoma model. The DISCO registry raised concern that dual antiplatelet therapy might be associated with worse outcomes than single antiplatelet therapy in conservatively managed SCAD (34). The interpretation remains limited by confounding, and subsequent commentary emphasized that observational data should not be treated as definitive evidence against DAPT in all SCAD patients (35). Randomized trials such as BA-SCAD are therefore critical because the question cannot be resolved by mechanistic intuition alone (36).

Biomarkers represent an early but important translational frontier. Differentiating acute SCAD from atherosclerotic AMI remains clinically difficult, particularly when angiographic findings are ambiguous. A circulating microRNA signature has been reported to distinguish acute SCAD from atherosclerotic AMI in discovery and validation cohorts (37). This work supports the feasibility of molecular diagnosis, but it is not yet ready for routine emergency use. Any biomarker must be validated in real-world chest pain populations, across time from symptom onset, across sex and ancestry, and in patients with overlapping diagnoses such as Takotsubo syndrome, myocarditis, or MINOCA.

Long-term prognosis studies continue to shape follow-up strategy. Multicenter observational data have shown that immediate and late outcomes vary by presentation, treatment strategy, and clinical context (38). Serial angiographic studies have also demonstrated that conservatively managed SCAD can evolve early, sometimes through progression of intramural hematoma, which reinforces the need for careful inpatient observation in selected patients (39). Symptom burden requires equal attention because migraine and other vasomotor phenotypes are common in SCAD cohorts and may reflect shared vascular biology, autonomic reactivity, or ascertainment effects (40).

Surgical revascularization deserves separate consideration because it may be lifesaving in selected high-risk presentations but remains supported mainly by case reports, small series, and retrospective experience. Coronary artery bypass grafting has been described for left main or proximal multivessel SCAD when ongoing ischemia, hemodynamic compromise, failed PCI, or threatened large myocardial territory makes conservative treatment unsafe (41). Pregnancy-related surgical reports highlight a distinctive limitation of CABG in SCAD: late competitive flow and graft narrowing may occur after spontaneous healing of the dissected native vessel (42). More recent case literature also illustrates emergent two-vessel CABG for critical distal left main SCAD identified during acute catheterization, reinforcing that surgery should be individualized through Heart Team assessment rather than treated as a routine pathway (43).

Genetic testing is another translational opportunity that requires discipline. A mechanism-informed approach would not recommend indiscriminate sequencing for every SCAD patient. Instead, genetics referral should be prioritized for patients with recurrent or multivessel SCAD, young age, family history, extracoronary aneurysm or dissection, syndromic connective tissue features, severe pregnancy-associated SCAD, or unusual vascular phenotypes. Polygenic risk may eventually contribute to counseling, but it currently lacks validated clinical thresholds. The immediate clinical value of genetics is highest when it identifies a pathogenic variant that changes surveillance, pregnancy counseling, trauma avoidance, family cascade testing, or blood pressure management.

Imaging follow-up should also be interpreted mechanistically. CCTA can document healing in selected patients, but its sensitivity depends on vessel size, lesion location, heart rate, and image quality. Intravascular imaging may clarify uncertain lesions but carries procedural risk and should not be used merely to satisfy curiosity. Brain-to-pelvis imaging helps identify FMD, aneurysm, dissection, or other arteriopathies, but findings should be stratified by severity and clinical relevance rather than presented as a binary positive or negative label.

**Figure 3:**
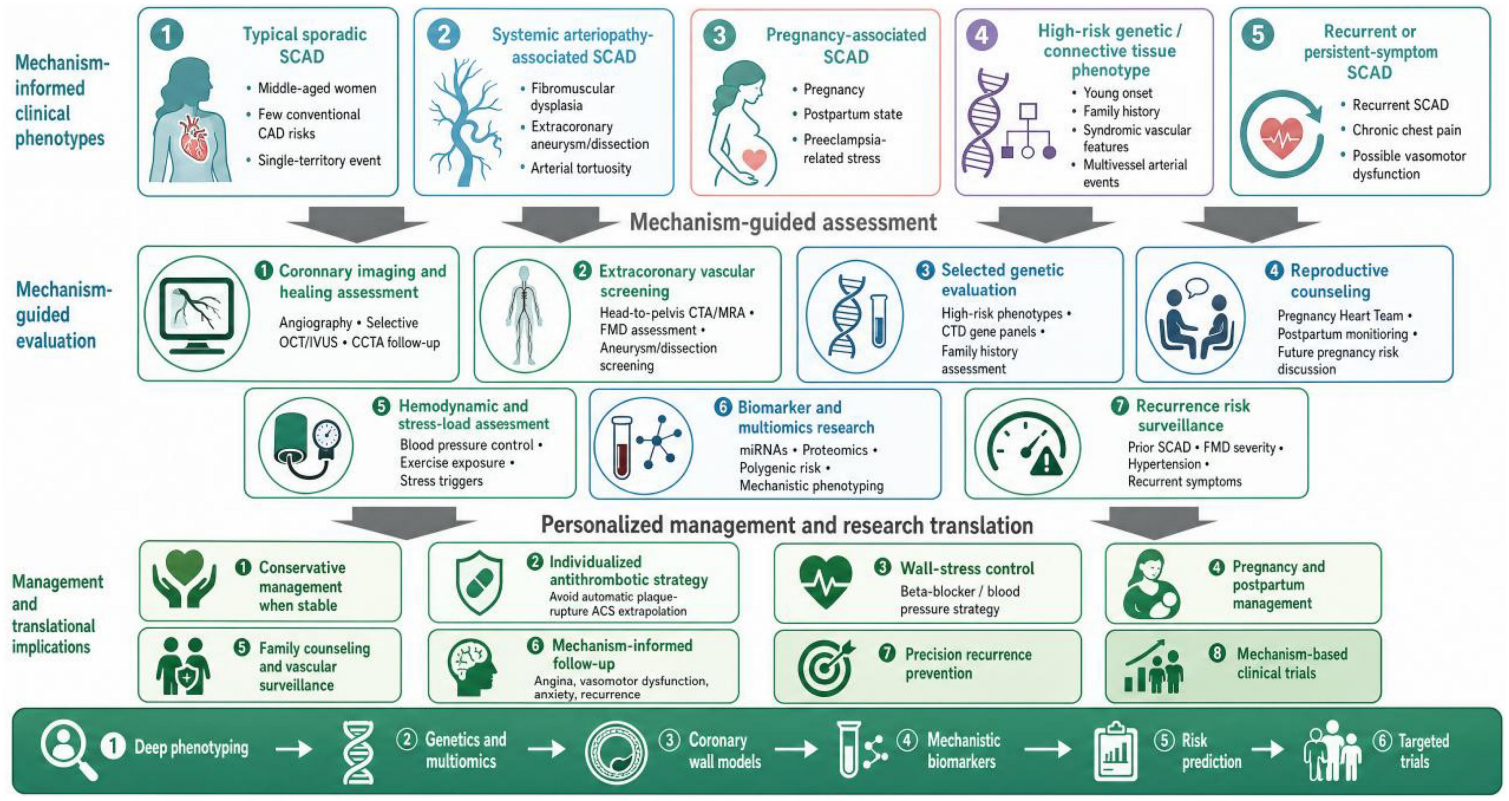
Mechanism-informed translational framework for spontaneous coronary artery dissection. This schematic illustrates how a mechanism-informed understanding of spontaneous coronary artery dissection (SCAD) can be translated into clinical phenotyping, targeted evaluation, individualized management, recurrence prevention, and future research. The upper tier categorizes patients into clinically recognizable but potentially overlapping phenotypes, including typical sporadic SCAD, systemic arteriopathy-associated SCAD, pregnancy-associated SCAD, high-risk genetic or connective tissue phenotypes, and recurrent or persistent-symptom SCAD. The middle tier links these phenotypes to mechanism-guided evaluation strategies, including coronary imaging and healing assessment, extracoronary vascular screening, selected genetic evaluation, reproductive counseling, hemodynamic and stress-load assessment, biomarker and multiomics research, and recurrence risk surveillance. The lower tier summarizes management and translational implications, emphasizing conservative management when stable, individualized antithrombotic strategy, wall-stress control, pregnancy and postpartum management, family counseling and vascular surveillance, mechanism-informed follow-up, precision recurrence prevention, and mechanism-based clinical trials. The bottom pipeline highlights the research trajectory required to move the field from descriptive phenotyping toward precision care: deep phenotyping, genetics and multiomics, coronary wall models, mechanistic biomarkers, risk prediction, and targeted trials. CAD, coronary artery disease; CCTA, coronary computed tomography angiography; CTD, connective tissue disorder; CTA, computed tomography angiography; FMD, fibromuscular dysplasia; IVUS, intravascular ultrasound; MRA, magnetic resonance angiography; OCT, optical coherence tomography; SCAD, spontaneous coronary artery dissection. The framework is intended to support individualized evaluation and research design; it should not be used as a validated decision algorithm requiring every patient to undergo every test or intervention.

The same wall-integrity model also provides a practical way to link biological heterogeneity to clinical action without treating all SCAD patients as a homogeneous population. As illustrated in Figure 3, mechanism-informed phenotypes can guide targeted evaluation, individualized management, recurrence surveillance, and enrollment into future mechanism-based studies. Table 3 complements this translational framework by ranking major clinical and research opportunities according to their evidence basis, present readiness, unresolved uncertainty, and the next study required before broader implementation.

**Table 3:** Translational opportunities and evidence gaps in SCAD.

Domain	Mechanism-informed strategy	Current actionability	Key uncertainty	Required next step
Acute diagnosis	Wall-aware angiography with selective intravascular imaging	Implemented in expert practice	When imaging benefit exceeds propagation risk	Prospective registry of ambiguous lesions with core-lab adjudication.
Acute treatment	Conservative management when stable and flow is preserved	Implemented in stable patients	Which anatomical subsets require early intervention	Morphology-outcome studies with standardized follow-up imaging.
Antithrombotic therapy	Individualize SAPT, DAPT, and anticoagulation according to PCI, thrombus, and IMH context	Evidence emerging	Causal effect of antithrombotic intensity	Randomized trials with healing and recurrence endpoints.
Wall-stress control	Blood pressure control and beta-blocker strategy when tolerated	Biologically plausible and commonly used	Magnitude of recurrence reduction and tolerability	Pragmatic trial with blood pressure variability and patient-reported outcomes.
Vascular screening	Head-to-pelvis CTA or MRA to assess FMD, aneurysm, dissection, and tortuosity	Implemented in many centers	How to stratify mild or incidental findings	Severity-based arteriopathy registry.
Genetic medicine	Selected genetics referral for high-risk phenotypes	Clinically useful in selected patients	Diagnostic yield across ancestry and phenotype	Genotype-phenotype-outcome studies with standardized return of results.
Reproductive counseling	Pregnancy Heart Team evaluation and postpartum monitoring	Expert-practice standard	True recurrence risk and modifiers	Prospective pregnancy-after-SCAD registry.
Biomarkers	miRNA, protein, and multiomic signatures for diagnosis and phenotyping	Research stage	Real-world diagnostic accuracy	Multicenter chest pain cohort with serial sampling.
Surgical revascularization	Heart Team consideration of CABG for left-main/proximal multivessel SCAD, failed PCI, ongoing ischemia, or hemodynamic compromise	Rare rescue option in selected high-risk cases	Case-based evidence and potential late graft failure after native-vessel healing	Registry-based assessment of surgical indications, conduit choice, graft patency, and long-term outcomes.

## 9 Key controversies and unresolved questions

The first unresolved question is whether inside-out and outside-in SCAD represent distinct molecular diseases or imaging manifestations of a shared wall-failure process. If they are distinct, future registries and trials should stratify by imaging evidence of intimal communication. If they are shared, the focus should shift toward common determinants of intramural hematoma expansion and healing.

The second controversy concerns FMD. FMD is clearly enriched in SCAD, but its role may range from causal arteriopathy to systemic marker to coincidental comorbidity depending on the patient. Resolving this requires standardized imaging protocols, blinded phenotype adjudication, genetic integration, and prospective outcomes rather than retrospective labels.

The third question is whether SCAD has molecular subtypes that can be recognized clinically. Candidate axes include matrix-dominant, vascular-tone-dominant, hemostasis-dominant, pregnancy-associated, and systemic-arteriopathy-associated SCAD. At present, these categories should be treated as testable hypotheses rather than clinical diagnoses.

The fourth controversy is antithrombotic therapy. Intensified antiplatelet or anticoagulant therapy may theoretically worsen wall bleeding, yet luminal thrombus and stented lesions may require antithrombotic treatment. The solution is not a universal rule but randomized and phenotype-aware evidence. Trial design should separate conservatively managed SCAD from PCI-treated SCAD and should capture lesion healing, recurrent SCAD, bleeding, and patient-centered outcomes.

The fifth question is how to counsel pregnancy after SCAD. Existing evidence is limited and potentially affected by selection bias because women who pursue pregnancy after SCAD may differ from those advised against pregnancy. Counseling should be individualized and multidisciplinary. Future registries should collect reproductive variables, assisted reproductive technology exposure, hypertensive disorders of pregnancy, lactation status, postpartum timing, imaging findings, genetics, and recurrence outcomes.

The sixth unresolved question is why SCAD recurs in some patients, often in different coronary segments. Recurrence implies persistent wall vulnerability, ongoing exposure to triggers, or both. Predictive models should combine clinical factors, FMD severity, coronary tortuosity, blood pressure variability, genetic susceptibility, pregnancy variables, and biomarkers. A purely culprit-lesion model will not be sufficient.

## 10 Future perspectives: toward a mechanism-informed taxonomy

The next generation of SCAD research should be organized around testable mechanisms rather than descriptive categories. Large prospective cohorts should collect standardized acute angiography, CCTA or vascular imaging, reproductive variables, blood pressure metrics, exercise and stress exposures, medication data, biospecimens, and long-term outcomes. These cohorts should be designed from the outset for mechanistic substudies rather than adding biology retrospectively.

Multiomic approaches are needed to connect genetic susceptibility to intermediate phenotypes. Whole-genome sequencing, transcriptomics, epigenomics, proteomics, metabolomics, and microRNA profiling should be integrated with imaging and outcomes. However, multiomics without a clear mechanism will generate noise. The priority should be to test whether matrix, wall cell regulation, vascular tone, local hemostasis, and systemic arteriopathy modules predict presentation, healing, recurrence, or treatment response.

Experimental systems must model the arterial wall rather than isolated cells alone. Patient-derived induced pluripotent stem cell VSMCs and fibroblasts can be exposed to cyclic strain, pressure, and matrix stiffness gradients. Three-dimensional vascular wall systems should include layered cell architecture and defined matrix composition, allowing investigators to test whether candidate variants alter mechanical failure, matrix remodeling, or response to adrenergic and endothelin stimuli. Microfluidic systems could incorporate blood components to study local hemostatic containment.

Pregnancy-associated SCAD should be treated as a mechanistic window. Longitudinal studies before conception, across gestation, and postpartum could measure vascular function, blood pressure variability, endothelial markers, matrix turnover, coagulation, inflammatory signatures, and reproductive exposures. Such studies are challenging but essential because pregnancy-associated SCAD may reveal biological thresholds that are not visible in nonpregnant patients.

Clinical trials should be mechanism-embedded. Trials of beta-blockers, antiplatelet intensity, and conservative versus invasive strategies should include lesion morphology, intramural hematoma burden, healing, recurrent lesion location, blood pressure variability, quality-of-life outcomes, and mechanistic biomarkers. Conventional cardiovascular endpoints alone will not be sufficient because SCAD is not an atherosclerotic plaque disease.

The field also needs diversity in ancestry and geography. Most genetic and clinical studies have been enriched for European ancestry and high-resource cardiovascular centers. A globally useful SCAD taxonomy must include Asian, African, Latin American, Middle Eastern, and Indigenous populations, as well as different healthcare systems. Cross-ancestry genetics may identify shared and population-specific risk loci and improve transferability of polygenic risk.

Building on the evidence hierarchy and translational framework above, a future SCAD taxonomy should be structured around lesion phenotype, systemic context, and modifiable stress environment rather than around a single descriptive label.

A practical future taxonomy may classify SCAD along three axes. The first axis is the proximal lesion phenotype: intimal communication evident, intramural hematoma-dominant without evident communication, occlusive, multivessel, or left-main/proximal high-risk disease. The second axis is systemic context: isolated sporadic SCAD, FMD-associated SCAD, pregnancy-associated SCAD, monogenic or syndromic arteriopathy-associated SCAD, and inflammatory or iatrogenically confounded mimics. The third axis is modifiable stress environment: hypertension or high blood pressure variability, intense isometric exposure, postpartum state, or medication-related bleeding risk. Such a taxonomy would be more useful than a single label because it links biology to decisions.

## 11 Conclusions

SCAD is entering a mechanistic era. The field has moved beyond the question of whether SCAD is a real and clinically important cause of myocardial infarction. The central question is now why the coronary arterial wall fails in specific patients and contexts. Current evidence is consistent with a model in which inherited susceptibility, matrix architecture, vascular wall cell regulation, vascular tone, local hemostasis, systemic arteriopathy, and trigger-related mechanical load converge on intramural hematoma formation. This model explains why SCAD differs from atherosclerotic ACS, why conservative management often succeeds, why antithrombotic therapy is uncertain, and why recurrence prevention requires more than plaque-focused cardiovascular prevention.

This framing also defines boundaries. SCAD should not be reduced to FMD, pregnancy, connective tissue disease, vasospasm, inflammation, or vasa vasorum rupture. Each may be relevant in subsets, but none currently explains the syndrome as a whole. The most productive path forward is a provisional, mechanism-informed taxonomy that recognizes heterogeneity at initiation and convergence at the level of coronary wall failure. Such a taxonomy could support better diagnosis, safer therapy, rational genetic testing, reproductive counseling, and targeted prevention. Achieving this will require prospective cohorts, experimental wall models, molecular biomarkers, cross-ancestry genetics, and clinical trials designed specifically for SCAD rather than adapted from atherosclerotic coronary disease.
